# Healthcare workers´ experiences and perceptions of the provision of health insurance benefits to the elderly in rural Tanzania: an explorative qualitative study

**DOI:** 10.1186/s12889-023-15297-4

**Published:** 2023-03-08

**Authors:** Paul Joseph Amani, Miguel San Sebastian, Anna-Karin Hurtig, Angwara Denis Kiwara, Isabel Goicolea

**Affiliations:** 1 Department of Health Systems Management, School of Public Administration and Management, Mzumbe University, Morogoro, Tanzania; 2 Department of Epidemiology and Global Health, Umeå University, Umeå, Sweden; 3 Department of Development Studies, Muhimbili University of Health and Allied Sciences, Dar Es Salaam, Tanzania

**Keywords:** Healthcare workers, Qualitative, Elderly, Health insurance, Rural, Tanzania

## Abstract

**Background:**

Healthcare workers play an important part in the delivery of health insurance benefits, and their role in ensuring service quality and availability, access, and good management practice for insured clients is crucial. Tanzania started a government-based health insurance scheme in the 1990s. However, no studies have specifically looked at the experience of healthcare professionals in the delivery of health insurance services in the country. This study aimed to explore healthcare workers’ experiences and perceptions of the provision of health insurance benefits for the elderly in rural Tanzania.

**Methods:**

An exploratory qualitative study was conducted in the rural districts of Igunga and Nzega, western-central Tanzania. Eight interviews were carried out with healthcare workers who had at least three years of working experience and were involved in the provision of healthcare services to the elderly or had a certain responsibility with the administration of health insurance. The interviews were guided by a set of questions related to their experiences and perceptions of health insurance and its usefulness, benefit packages, payment mechanisms, utilisation, and availability of services. Qualitative content analysis was used to analyse the data.

**Results:**

Three categories were developed that describe healthcare workers´ experiences and perceptions of delivering the benefits of health insurance for the elderly living in rural Tanzania. Healthcare workers perceived health insurance as an important mechanism to increase healthcare access for elderly people. However, alongside the provision of insurance benefits, several challenges coexisted, such as a shortage of human resources and medical supplies as well as operational issues related to delays in funding reimbursement.

**Conclusion:**

While health insurance was considered an important mechanism to facilitate access to care among rural elderly, several challenges that impede its purpose were mentioned by the participants. Based on these, an increase in the healthcare workforce and availability of medical supplies at the health-centre level together with expansion of services coverage of the Community Health Fund and improvement of reimbursement procedures are recommended to achieve a well-functioning health insurance scheme.

**Supplementary Information:**

The online version contains supplementary material available at 10.1186/s12889-023-15297-4.

## Background

Healthcare systems in low- and middle-income countries have to adapt to the needs of a rapidly increasing elderly population [1]. In these countries, the majority of this group is commonly socio-economically vulnerable, has poorer health that requires more healthcare visits, and reside in rural areas where healthcare infrastructure is more limited than in urban settings [2]. In order to address these challenges, a series of healthcare reforms, often including public health insurance (HI) schemes, has been underway in the last two decades, with the aim of enhancing access to care and promoting economic protection to the elderly [3].

Government-based HI has been established as a risk-sharing mechanism, particularly in Sub-Saharan Africa (SSA), to finance healthcare, minimise social inequality, and enable access to care, particularly to socially disadvantaged populations [4–7]. Overall, experience has shown that HI not only increases access to and utilisation of healthcare but also extends financial protection to an economically vulnerable population, such as the elderly [8]. The reported success behind HI strongly supports the goal of achieving universal health coverage, as it focuses on creating a healthcare system that is able to provide equitable access to healthcare for all [9]. Nevertheless, HI has also been criticised for not being able to meet the expectations of the insured regarding quality of care, to reduce waiting times, and to financially protect vulnerable population groups equally [10–12].

Healthcare workers play an important part in the delivery of HI benefits, and their role in ensuring service quality and availability, access, and good management practice for the insured clients is crucial. Their role extends to translating the insurance policy into medical practice, an area which requires not only unique knowledge and skills but also experience [13]. In addition to their involvement in providing care, administratively healthcare personnel should work to create a conducive environment that responds to the health needs of the insured community and act as a link between patients and insurance schemes for billing and payment [14].

Literature addressing healthcare workers’ perceptions of the functioning of HI in SSA is scarce. In a study conducted in both Kenya and Ghana, health service providers reported how delayed reimbursement by insurance schemes negatively affected the ability of the facilities to restock medicines and pay bills [15]. In another study from Ghana, providers considered HI as a revenue source to finance facility activities, but complained about the failure of the scheme to provide reimbursement on time and ensure service availability, which influenced some providers to prioritise patients who could make cash payments [16]. Shortage of medicines and delayed reimbursement have also been reported by service providers in the SSA context of HI [17].

In Tanzania, the first attempt to implement HI started with the Community Health Fund (CHF), which focused on rural areas. The CHF was first piloted in Igunga district in 1996 and was later rolled out to the other districts in the country. On average, whereas the CHF has managed to extend the coverage to 24% of the population, below the initial 30% target by 2015, only 6% are covered under the National Health Insurance Fund (NHIF) [9, 18]. In principle, the CHF operates as a voluntary rural-based scheme dependent on household enrolment of up to six members who obtain a yearly single membership card that can be used in accessing primary and secondary health facilities within the district jurisdiction. Recognising the usefulness of HI, in 2001 the government also established the NHIF, a compulsory scheme for government sector employees, which was later extended in 2013 to the informal sector as a voluntary insurance [19]. Although these two schemes operate in parallel in each district, they differ in their financial capacity and services offered. In contrast to the CHF, the NHIF provides coverage for a wider range of benefits at all healthcare system levels and across the country. These efforts, in line with both the Tanzanian health policy 2015 and the African agenda 2063, aim to improve not only access to but also the availability and affordability of quality public healthcare services [20].

One of the specifically targeted populations of the CHF has been the elderly, whose ability to pay for healthcare is limited. Currently, 6% of the total Tanzanian population constitutes people above 59 years of age, and this percentage is projected to increase to 11% by 2050 [21]. Since the majority of this population reside in rural areas, where availability and access to social services including healthcare are importantly needed but still very limited, the introduction of the CHF was seen as an indispensable mechanism to provide the elderly with access to affordable and quality care [20].

Available research from Tanzania has shown that the CHF can reach socio-economically disadvantaged populations and thus improve their access to healthcare [20, 22–25]. When it comes specifically to the elderly, our own study has shown that HI has the potential to improve the utilisation of healthcare among rural elderly residents [9]. Nevertheless, studies have also identified operational challenges in implementing HI, such as mistrust [20], a limited benefit package and delayed reimbursement [20, 24], poor quality care, the inability to pay membership fees, and an unclear exemption policy [26, 27].

Even though a number of studies have been conducted to explore the contribution of HI to the Tanzanian population [9, 19, 28], to our knowledge, no studies have specifically looked at the healthcare workers´ views on the delivery of HI services in the country. In order to fill this gap, this study aimed to explore healthcare workers’ experiences and perceptions of the provision of HI benefits for the elderly in rural Tanzania.

## Methods

### Study setting

This study was conducted in the districts of Nzega and Igunga in Tabora region, central Tanzania. Tabora region is home to a population of about 2.3 million, of which 901,979 (Nzega is 502,252 and Igunga 399,727) people reside in the study area [29] and approximately 50,547 (6%) constitute people aged 60 years and above. Sukuma and Nyamwezi are the two major ethnic groups in the region. While the literacy status for persons aged 15 years and above stands at 59% in the region, it is 56.1% for Nzega and 58.7% for Igunga. About 80% of the region’s wealth comes from agriculture, which involves around 76% of the population. Tobacco and cotton are mainly grown for cash markets, whereas maize, sorghum, cassava, and sweet potatoes constitute the main food crops. Nzega is divided into 37 wards with 151 villages and Igunga into 26 wards with 93 villages. In these districts, there are about 5,600 (6% in Nzega and 5% in Igunga) elderly aged 60 and above who are enrolled with the CHF and NHIF schemes [9]. The districts were chosen because Igunga is the first district in the country where HI was implemented, and both are rural districts.

### Health insurance at the district level

Financing healthcare at the district level relies on a yearly approved government budget. This budget comes from central government allocations (mainly from taxes), grants from development agencies, the local government authority’s own sources, user fees, and contributions from HI schemes. The CHF operates at the district level under a CHF coordinator whose responsibility is to track the membership levels and manage the funds in the district. The office also works with ward officers to motivate households to join the scheme. The CHF funding depends partly on membership premiums (USD 2.18–6.55 per household per year set by the district health administration) and partly on a central government subsidy that equals the amount contributed per household. These funds are deposited in the respective district CHF account, and individual health facilities are required to make reimbursement requests to these offices [22]. The distribution of funds is based on the district health plan’s directive as approved by the responsible local government. Experience from the rural districts has shown that the CHF funds are spent on the following: about 71% to purchase medicines and supplies; 18% to support facility rehabilitation and repair; and 11% to purchase receipt books and print CHF cards [30]. Delays, particularly from the government budget and long administrative procedures of the CHF, are common, contributing to a shortage of funds in the facilities. Once registered, each household member is given a renewable health card that entitles the member to a basic package of healthcare services within the district’s health facilities throughout the year.

The NHIF operates at the regional level and is mainly funded through a member contribution of 6% of the employee’s monthly gross salary that is evenly shared between employer and employee. Service providers across the region submit annual requests for repayment for the services offered to NHIF clients in the previous year to the NHIF regional offices. The NHIF coordinator at the facility is responsible for this assignment. Reimbursement is made according to fees for services provided depending on the type of healthcare facility level.

### Study participants

This study involved a variety of healthcare workers employed at both primary and secondary health facilities who were purposively selected considering the following inclusion criteria: at least three years of working experience at the facility, involvement in the provision of healthcare services to the elderly, and/or having a certain responsibility with administration of HI. A total of 11 primary healthcare facilities (nine health centres and two district hospitals) were selected for the study. At each facility, the person in charge suggested one candidate to participate in the study based on the inclusion criteria. From the 11 invited participants, eight showed up for the interviews.

The sample consisted of an equal number of males and females whose ages ranged between 38 and 59 years (see Appendix 1). They differed in terms of their working tasks: two community development officers working also as CHF and NHIF coordinators; two health system administrators, responsible for the day-to-day management of the health facilities; two clinical officers, attending elderly people regardless of insurance status; and two medical doctors, in charge of the medical services at the hospital level.

### Data collection

We conducted eight in-depth interviews with healthcare providers using an interview guide that was inspired by the results of our earlier studies in the same setting. The guide was revised after being piloted with two persons who did not form part of the study group. All participants received an invitation to a face-to-face interview at an agreed time and place between November 2018 and March 2019. The interviews were conducted by the first author, in the providers’ free time, mostly after office hours in an available consulting room or an interviewee’s office at their respective healthcare facility. The interviewer made efforts to facilitate the interviewees so that they felt free to express their opinions during the study.

Each interview was audio-recorded and lasted for approximately 35–55 min. The interviews were guided by a set of questions that aimed at promoting discussion regarding healthcare workers’ experiences and perceptions of providing the benefits of HI to the insured elderly. The issues that were at the centre of the interview included workers’ experiences of HI and their perspective of its usefulness, benefit package, payment mechanisms, utilisation, and availability of services for the insured elderly. During the interview, probes were used to motivate discussion, foster understanding, and explore new topics from their experiences. All interviews were conducted in ‘Swahili’, which was the local language of the interviewer and the participants.

### Data analysis

Data analysis started by transcribing the audio-taped interviews. In order to ensure accuracy of the transcripts, the first author cross-checked the transcripts back to back with the audio responses. The transcripts were later translated into English to facilitate analytical discussions within the research group. We used qualitative content analysis as proposed by Graneheim and Lundman [31] to analyse the texts from the transcripts. The process began by reading the transcripts to familiarise ourselves with the material. Afterwards, the verbatim transcripts were condensed to develop condensed meaning units. The next steps consisted of coding, building codes into categories, and making analytical interpretive connections between categories [32]. The first author (PA) developed the first coding and then worked with IG to further develop groups of codes and categories. Several rounds of meetings were held between all the authors to discuss the categories (labels and content) and establish connections between them. We used the freely available Open-code software 4.03 to support the analysis, particularly the coding process [28].

### Ethical considerations

The research protocol was approved by the research and ethics committee of the Muhimbili University of Health and Allied Sciences, Dar es Salaam, Tanzania in May 2017 (reference no. 2017–05-24/AEC/Vol.XII/70). Permission for data collection was obtained from the District Executive Directors of Igunga and Nzega districts. The participants were briefed on the purpose of the study before conducting each interview, and written consent was obtained from all participants. They were informed that participation in the study was voluntary and that they could withdraw whenever they felt it necessary. In addition, they were also informed that the interview transcripts would be used for academic research and publication. Finally, participants were notified that their quotations would be used in the manuscript. We assured participants that confidentiality would be maintained, as the transcripts would be read only by the research team and would be presented in a manner that would preserve anonymity.

## Results

Three categories were developed that describe healthcare workers’ experiences and perceptions on delivering the benefits of HI for the elderly living in rural Tanzania. Participants perceived that HI was an enabler of access to healthcare, promoting equitable utilisation; however, a shortage of necessary resources and lack of funds due to delayed reimbursement affected service availability and delivery.

### Health insurance is an enabler of equitable access to healthcare

Throughout the interviews, participants agreed that HI was a useful and important means of facilitating access to the healthcare system, particularly for the elderly. It was often mentioned that HI acted as an enabler of access to free healthcare, reducing the risk of the elderly not seeking care when needed and therefore contributing to avoiding future poor health outcomes, including prolonged illness, disability, and even mortality:

*It enables the elderly to get proper treatment at a hospital apart from going to buy tablets at a medical store without being diagnosed or given a prescription…this [self-medication] may lead to continued pain due to an inappropriate cure that may result in death, which could have been prevented.* (Health system administrator)

According to the participants, HI facilitated access mainly through the elimination of financial barriers to access the facilities because out-of-pocket payments were no longer needed. As one interviewee expressed:

*Those with HI…receive free treatment, including consultation, laboratory tests, and medicine…and it is convenient for the elderly together with their family.* (Insurance coordinator)

*The health insurance card helps the elderly to get treatment without any payments…whether they are to be admitted or treated on a daily basis…they do not need to have money to get treatment.* (Health system administrator)

Since the utilisation of services was based on the need for care, not on the ability to pay, equity in access was acknowledged as an important contribution of HI. One of the participants summarised it in this way:

*All with insurance are treated the same…they are able to access services, to be seen by a doctor, get laboratory tests, and receive medicines, those that are available, as they reach the hospital.* (Insurance coordinator)

In the previous quotation, the insurance coordinator highlighted the equitable access to those resources but only to ‘those that are available’, pointing out a caveat that HI may not be able to tackle. This and other related challenges are discussed in the following categories.

### Shortage of non-financial resources affects service delivery

The shortage of non-financial resources experienced by participants was discussed as an important challenge to provide adequate HI benefits to the elderly. In all the interviews, the interviewees talked about how the lack of human resources in both numbers and skills not only affected the provision of quality healthcare but also increased the long waiting time for the insured elderly:

*Most of our facilities have less than 60% of the required workforce, and the burden is much felt in these rural areas, mostly in public hospitals…no geriatricians.* (Medical doctor)

*When they [insured elderly] come, they want to get services without any delay and leave the facility [as soon as possible]…however, they have to wait long before the doctor sees them due to shortages of staff.* (Health system administrator)

Similarly, participants complained about the frequent scarcity of medicines and laboratory services despite the fact that they should be provided free of charge to the insured. The unavailability of these services implied an extra cost for the elderly with HI because they were forced to buy them from private drugstores and laboratories:

*There is a lack of medicines for insured elderly with the CHF, especially nerve related, arthritis, diabetes, and pressure, which mostly are scarce…they are not enough……instead, they have to go and buy…for NHIF members, there are arrangements for them to get the missing prescriptions from a pharmacy without being required to pay.* (Clinical officer)

*Due to a lack of ultrasound at our facilities, the elderly face that problem, although they are supposed to be given services using both exemption and the CHF…they have to get it somewhere else, in most cases through their own payment…while those with the NHIF obtain a form to get the medicines from an accredited facility.* (Medical doctor)

As the previous quotes highlight, participants perceived that access to free drugs and tests was more challenging for the elderly with the CHF than for those with the NHIF, who could get them free of charge from private sources when they were not available within public healthcare facilities. Participants were also aware of the problems originating from the restricted benefit package under the CHF, which limits the service coverage outside the district of registration. Any attention beyond the district, even in public healthcare facilities, would imply an extra cost. As one of the participants expressed it:

*The CHF covers very little and has a very long referral process, which causes most of the elderly to despair or end on incomplete treatment, because they are supposed to report first at the dispensary, and thereafter they can go as far as to the district level only...when they travel to other districts, they will have to pay.* (Clinical officer)

Even though a shortage of resources in the healthcare facilities impeded the provision of HI benefits, such a limitation affected those with the CHF more compared to those with the NHIF. In addition, lack of funds due to delayed reimbursement, as evidenced in the coming section, affected the availability of services, especially ones needed by those with HI.

### Delayed reimbursement affects service availability

The availability of funds was discussed in detail by the participants as another challenge affecting the provision of healthcare services to the insurance beneficiaries. Overall, managers acknowledged that HI constituted an important part of healthcare financing and experienced that the refund process had improved over the years. As the insurance coordinator explained:

*If the health facility has clearly documented and filled the claim forms appropriately, they are reimbursed without much delay.* (Insurance coordinator)

However, there were also experiences of frustration, as the insurance scheme often took a long time to refund the facilities for the services rendered to their beneficiaries. One medical doctor reflected, ‘It is like a dream to be paid our claims on time’, whereas an insurance coordinator added, ‘The refund process is always delayed and takes a long time’.

This delay contributed to making the health system poorer, affecting the availability of resources needed for appropriate healthcare delivery and increasing the uncertainty of managers for planning and budgeting purposes:

*Delay of reimbursements causes unstable provision of health services, for example, medicines and other hospital equipment.* (Insurance coordinator)

*It may even take two to three months to receive payments. This leads to poor services because we do not have money to make service replacements.* (Health system administrator)

Two additional constraints of the HI system were discussed in relation to delayed reimbursement. The first referred to the complexity of the current HI reporting system. As two respondents mentioned:

*It is taking much time to prepare the claims and also much time to wait for the payments.* (Insurance coordinator)

*The process of preparing claims is not easy, and sometimes it goes back and forth and requires clarity and sufficient evidence matching the payment being requested...failure to do this may cause the facility to lose income.* (Insurance coordinator)

In the second constraint, a shortage of healthcare workers with limited capacity regarding claims management was experienced as a barrier to making the process faster. Furthermore, claims that were not submitted correctly would contribute to delaying the reimbursement. As two participants mentioned:

*It takes a long time to prepare the claims because healthcare providers [staff] are few and require training regarding claims management.* (Health system administrator)

*If the health facility has not clearly documented and filled the claim forms appropriately, they are not reimbursed on time.* (Insurance coordinator)

As presented above, the delay in reimbursement not only frustrated healthcare workers but also affected the financial security and therefore the availability of the expected healthcare.

## Discussion

Since the introduction of HI in Tanzania, no studies have been conducted focusing on the experiences and perceptions of healthcare workers as key stakeholders in the delivery of HI benefits to the elderly population. This study has shown that healthcare workers perceived HI as an important mechanism to increase healthcare access for the elderly. However, alongside the provision of HI benefits, several challenges were experienced, such as a shortage of human resources and medical supplies as well as operational issues related to delays in funding reimbursement. In the following section, we discuss our findings in relation to increased healthcare access, the consequences of a shortage of resources, and the structural challenges of the current HI system.

### Increased healthcare access

From the perspective of the healthcare workers, HI has become an important enabler of healthcare access and utilisation by the elderly due to the elimination of out-of-pocket payments as a prerequisite for access to healthcare. This finding corresponds to the aim of HI in protecting vulnerable groups against healthcare costs. Their perception aligns with findings from our previous research in the same setting, which showed an increase in utilisation of both inpatient and outpatient care among the insured compared to the uninsured elderly [9]. Similarly, other studies from Tanzania [23, 33] and elsewhere [34] have pointed in the same direction. For example, a study exploring the views of one rural community regarding the CHF [24] reported HI as being well accepted as an alternative mechanism to reduce out-of-pocket payments. Several other studies from SSA have also shown that HI has the potential to provide risk protection, thereby reducing the burden of healthcare costs among the poor [6] and making it a key mechanism for achieving universal health coverage [35].

Due to this financial protection, the healthcare workers also acknowledged HI as a promoter of equitable access based on needs, not socio-economic status. In the same line, our own earlier study from the same area showed that elderly people with HI were able to use more outpatient and inpatient healthcare, for the same needs, compared to their non-insured counterparts [9]. Our findings, however, are different from insurance studies from Ghana and Burkina, where inequitable access was evident, as the richest seemed to have better access to HI and its benefits than the more vulnerable populations [36, 37].

Furthermore, access to care was perceived to be more challenging for those insured by the CHF compared to the NHIF. Comparatively, CHF contributions are low and thus influence the number of insurance benefits that can be covered. The number of services they access is limited to primary healthcare facilities, most of which are rurally based and are inadequately equipped for essential care. This finding is in line with a study by Mtei [19] from Tanzania, which reported that healthcare benefits to CHF members is determined by the amount of money they contribute per household.

### Shortage of resources

Interviewees also highlighted the failure of the system to deliver important insurance benefits due to the experienced shortage of significant resources, including healthcare workers in both number and skills, as well as medicines and laboratory services. A lack of healthcare providers is prevalent in Tanzania, particularly in rural areas. Studies have shown that government facilities suffer a shortage of over 67% of their required levels of medical doctors in the country [38, 39]. This scarcity, particularly at the primary healthcare level, impinges on the ability of these facilities to serve patients adequately, especially when they come in large numbers [40]. It may, in addition, influence insurance services to be perceived as poor and insufficient to meet the expectations of insurance recipients and thus lead to a low uptake of HI [24, 41].

Studies looking at the implementation of HI in rural Tanzania have reported that many healthcare facilities commonly experience a lack of medicines due to delayed deliveries from central medical store departments [41] and a scarcity of funds to purchase medicines and other basic medical supplies [26]. This shortage of basic supplies at public healthcare facilities forces the insured to pay for drugs and laboratory services at private pharmacies [27]. These additional payments, despite having a CHF, may lead to distrust and low uptake of HI as well as a delay in seeking healthcare on time [24].

### System failures

Two additional structural issues that affected HI functioning were experienced by the participants, especially managers: inadequate funds to finance healthcare and delays in the reimbursement process from the insurance schemes. In practice, the functioning of the Tanzanian health system is decentralised and follows the subsidiary principle that places service delivery responsibility at the lowest government level closer to the community. Starting in 2000, the Direct Health Facility Financing was introduced under the Regional Administration and Local Government to enhance fiscal decentralisation in the health sector, with a focus on improving primary healthcare services [42]. Findings from a study from Tanzania have reported inadequate funding due to low budgetary allocation to the health system [43], which challenges the availability of healthcare, especially in rural areas. These results are similar to those from other countries in SSA whose healthcare budgets are hampered by an inability of the governments to finance healthcare [44, 45].

Our findings also showed that delayed reimbursement can affect service delivery for two possible reasons: some facilities might not be completing claims on time due to lack of providers’ understanding of the process and insurance schemes delaying payments due to bureaucratic procedures. This delayed reimbursement goes against the requirements of the HI Act of 2001, which states that repayment of claims should be settled within 60 days after being submitted [46]. A practice in the rural districts is that all the funds are first pooled into the district-level CHF account, and the health facilities are required to send requests to the districts [22]. However, a shortage of staff at the facility level makes the compilation process even slower [47]. Verification procedures at the district level may delay the process further [22]. Similarly, a study from Ghana, which focused on exploring the challenges in provider payments under the National Health Insurance Scheme, reported delayed reimbursement because of failure of the provider to prepare verifiable claims due to a shortage of manpower [48]. Another study from the same country reported that reimbursement was delayed by between three to six months, increasing the difficulties of the facilities to complete the stocking of medicines [49].

### Methodological considerations

The informants were purposely chosen to provide a variety of experiences and perspectives on the challenges to providing the benefits of HI to the elderly in this rural setting. The trustworthiness of the study was enhanced in several ways. We used an interview guide that was developed based on the issues that emerged from our earlier studies from the same area. Quotations extracted from the scripts aimed to illustrate that our interpretations were grounded on the data and thus increased the credibility of the study. Since the interviews were conducted in Swahili and later translated into the English language to allow the participation of the other authors, it is possible that some meanings from the original narration got lost in the translation process. However, the involvement of the first author in the interviews, who is a native speaker and familiar with the setting where the respondents are based, increased the credibility of the study findings. We also described the characteristics of the context where the study took place so that the reader can judge how transferable our findings are to similar rural settings. Since the study included respondents from eight health facilities from the study districts, the findings may not represent the situation from other districts and the country in general. Nevertheless, these facilities can serve as a representative sample for the public primary healthcare system in the country.

## Conclusion

This study explored the healthcare workers´ experiences and perspectives on the delivery of HI services to the elderly in rural Tanzania. Participants in these rural districts perceived that HI is an important and significant enabler of access to care for the elderly. However, they also experienced a shortage in human and material resources at the healthcare facility level and delays in reimbursement as main limitations for a well-functioning HI system. Considering the experiences of the healthcare workers, it is the authors’ view that several efforts should be made by the government to ensure that HI functions well. The smaller health service coverage of the CHF when compared to the NHIF needs to be revisited in order to create a fairer scheme. Strategic health system reforms are needed nationally, not only related to HI but also regarding the demand and distribution of the healthcare workforce and availability of medicines and medical equipment at the facilities, particularly in rural areas. The management of the HI would require further restructuring in order to facilitate the reimbursement procedures at all levels. Finally, further research is recommended to assess the implementation of HI in urban areas and to evaluate interventions targeting the improvement of HI in the context of current national health reforms.

## Supplementary Information

**Additional file 1: Appendix 1.**Characteristics of the respondents.

## Data Availability

The dataset that was generated and/or analysed during the current study is not publicly available owing to protect the confidentiality of the respondents, but are available from the corresponding author on reasonable request.

## Abbreviations

CHF: Community Health Fund
DHFF: Direct Health Facility Financing
HI: Health Insurance
LGAs: Local Government Authority
NHIF: National Health Insurance Fund
SSA: Sub-Saharan Africa
