# Supplementary material for: Healthcare workers´ experiences and perceptions of the provision of health insurance benefits to the elderly in rural Tanzania: an explorative qualitative study
Source: BMC Public Health. 2023 Mar 8;23:459. doi: 10.1186/s12889-023-15297-4 (PMC9996914; doi:10.1186/s12889-023-15297-4)
Supplement: Supplementary file 1 — Additional file 1: Appendix 1.Characteristics of the respondents. [file 12889_2023_15297_MOESM1_ESM.docx]

***Healthcare workers´ experiences and perceptions of the provision of health insurance benefits to the elderly in rural Tanzania: An explorative qualitative study***

**Appendix**

Appendix 1. Characteristics of the respondents

| S/n | Age | Gender | Profession | Position |
| --- | --- | --- | --- | --- |
| 1 | 45 | Woman | Community development officer | CHF coordinator |
| 2 | 36 | Woman | Health system administrator | Hospital administrator |
| 3 | 59 | Man | Clinical officer | Doctor for the elderly |
| 4 | 38 | Man | Medical doctor | Facility in charge |
| 5 | 56 | Man | Clinical officer | Doctor for the elderly |
| 6 | 45 | Woman | Community development officer | NHIF coordinator |
| 7 | 46 | Man | Medical doctor | Facility incharge |
| 8 | 43 | Woman | Health system administrator | Hospital administrator |
